# The Interplay of Variants Near *LEKR* and *CCNL1* and Social Stress in Relation to Birth Size

**DOI:** 10.1371/journal.pone.0038216

**Published:** 2012-06-07

**Authors:** Anokhi Ali Khan, Alina Rodriguez, Sylvain Sebert, Marika Kaakinen, Stéphane Cauchi, Philippe Froguel, Anna-Liisa Hartikainen, Anneli Pouta, Marjo-Riitta Järvelin

**Affiliations:** 1 Department of Epidemiology and Biostatistics, Medical Research Council Health Protection Agency Centre for Environment and Health, Imperial College London, London, United Kingdom; 2 Medical Research Council Social Genetic Developmental Psychiatry Centre, King’s College London, London, United Kingdom; 3 Department of Social Sciences –Psychology, Mid Sweden University, Östersund, Sweden; 4 Institute of Health Sciences, University of Oulu, Oulu, Finland; 5 Biocenter Oulu, University of Oulu, Oulu, Finland; 6 Unités Mixte de Recherche 8199, Centre National de la Recherche Scientifique, Institut de Biologie de Lille, Université Lille 2, Institut Pasteur, Lille, France; 7 Genomic Medicine, Hammersmith Hospital, Imperial College London, London, United Kingdom; 8 Department of Clinical Sciences/Obstetrics and Gynecology, University of Oulu, Oulu, Finland; 9 National Institute for Health and Welfare, Oulu, Finland; John Hopkins Bloomberg School of Public Health, United States of America

## Abstract

**Background:**

We previously identified via a genome wide association study variants near *LEKR* and *CCNL1* and in the *ADCY5* genes lead to lower birthweight. Here, we study the impact of these variants and social stress during pregnancy, defined as social adversity and neighborhood disparity, on infant birth size. We aimed to determine whether the addition of genetic variance magnified the observed associations.

**Methodology/Principal Findings:**

We analyzed data from the Northern Finland Birth Cohort 1986 (n = 5369). Social adversity was defined by young maternal age (<20 years), low maternal education (<11 years), and/or single marital status. Neighborhood social disparity was assessed by discrepancy between neighborhoods relative to personal socio-economic status. These variables are indicative of social and socioeconomic stress, but also of biological risk. The adjusted multiple regression analysis showed smaller birth size in both infants of mothers who experienced social adversity (birthweight by −40.4 g, 95%CI −61.4, −19.5; birth length −0.14 cm, 95%CI −0.23, −0.05; head circumference −0.09 cm 95%CI −0.15, −0.02) and neighborhood disparity (birthweight −28.8 g, 95%CI −47.7, −10.0; birth length −0.12 cm, 95%CI −0.20, −0.05). The birthweight-lowering risk allele (SNP rs900400 near *LEKR* and *CCNL1)* magnified this association in an additive manner. However, likely due to sample size restriction, this association was not significant for the SNP rs9883204 in *ADCY5*. Birth size difference due to social stress was greater in the presence of birthweight-lowering alleles.

**Conclusions/Significance:**

Social adversity, neighborhood disparity, and genetic variants have independent associations with infant birth size in the mutually adjusted analyses. If the newborn carried a risk allele rs900400 near *LEKR/CCNL1*, the impact of stress on birth size was stronger. These observations give support to the hypothesis that individuals with genetic or other biological risk are more vulnerable to environmental influences. Our study indicates the need for further research to understand the mechanisms by which genes impact individual vulnerability to environmental insults.

## Introduction

Being born small is associated with increased risk of perinatal morbidity and hospitalization [1], [2], poorer developmental and cognitive outcomes in childhood [3], [4], as well as cardiovascular disease, non-insulin dependent diabetes and intermediate risk factors for chronic diseases in adulthood [5]–[11]. Examining factors associated with small birth size is important to improve our understanding concerning the links between disturbed fetal growth and the development of disease later in life.

We recently identified in a large-scale genome wide association study (GWAS) that variants near *LEKR* and *CCNL1* and in the *ADCY5* are associated with birthweight [12]. Better understanding of the interplay between genetics and environmental factors would strengthen our ability to predict outcomes. Various environmental factors including social stress characterized by low social class, social adversity, or social disparity, have been linked to small birth size, though results are inconsistent [13]–[17]. This inconsistency may be due to methodological differences across studies concerning measurement of social factors, insufficient statistical power, and inclusion of covariates. Whether genetic variance adds to the association between social stress and birth size has not been previously studied.

Neighborhood environment is also known to be associated with lower birthweight and influence morbidity and mortality [18]–[20], independent of socio-economic status (SES). Neighborhood social disparity, *i.e.* living in areas where neighborhood financial capacity differs from individual SES, is associated with higher all-cause mortality [17]. This disparity may be explained partly through differences in access to care and amenities between neighborhoods as well as social stress. However, previous studies, examining the association between neighborhood social disparity and birth size, have not been able to consider important covariates such as smoking, maternal pre-pregnancy BMI, and ethnicity, thus potentially biasing results [18]–[20]. The interplay of genetic determinants with regard to neighborhood social disparity and birth size is unknown.

Our primary objective was to examine whether social stress and variance in the previously identified birthweight-lowering alleles would contribute in an additive manner to birth size (birthweight, birth length, head circumference and ponderal index). We used data from the Northern Finland Birth Cohort 1986 (NFBC 1986) and operationalized social adversity as the presence of at least one known environmental factor associated with stress at the individual level [21]–[24]. We used young maternal age, an indicator of poor social conditions and behavioral risk factors [25]; low education, an index of social class [26]; and single parenthood, associated with low household income and lack of social support [27]. These three indicators of social adversity have each been previously associated with low birthweight [25], [27]–[40] and poor developmental outcomes [24]. We hypothesized, based on potential biological vulnerability, that there is an association between social stress (individual social adversity or neighborhood social disparity) during pregnancy and smaller infant size at birth. In addition we hypothesized that this association will be magnified in individuals carrying birthweight-lowering alleles near *LEKR* and *CCNL1* or in *ADCY5*.

## Materials and Methods

### Study Cohort

The current study is based on data from the NFBC 1986 cohort, which comprises 9362 pregnant women (99% of pregnant population) and 9203 live-born singletons with expected date of birth between July 1985 and June 1986 from the provinces of Lapland and Oulu in Finland [41], [42].

Data concerning maternal health and social-demographics were collected via medical records, examinations/interviews by midwives, and data from a self-report questionnaire administered to pregnant women at the first visit to maternity health centers, approximately gestational week 12 and returned by gestational week 24 if still pregnant [41]. This study was approved by the ethics committee of the University of Oulu in accordance with the Declaration of Helsinki.

We had information on maternal social adversity for 9106 mothers and genotype for 5369 children [12].

### Assessment of Social Stress

Social stress at the individual level consisted of the sum of three stressors, thus scores ranged between 0 and 3. Young maternal age was defined as being <20 years at time of birth and coded as 1, otherwise as 0 [25]. Low education was coded as 1 when maternal education was <11 years or 0 if higher [26]. Unmarried maternal marital status was coded as 1 if single, divorced or widowed and 0 if married or cohabiting with the expectant father [27].

Social stress at the neighborhood level, i.e. neighborhood social disparity, was defined as a discrepancy between the family SES and the neighborhood financial estimate. A disparity score was created by comparing family SES (highest maternal or paternal occupation) with neighborhood financial estimate. Maternal and paternal occupation were categorized as 1 = professional, 2 = upper white collar, 3 = lower white collar, 4 = unskilled worker, 5 = farmer/farmer’s wife owning >8 hectares of land, 6 = farmer/farmer’s wife owning <8 hectares of land. Neighborhood financial estimate was based on financial capacity category (FCC) of the neighborhood for the 1982–92 classification by the National Finnish KOUTA database and rated from one (deprived) to six (affluent) [41], [43]. FCC takes into account factors such as density and age distribution in the population, income, expenditure on social and health care, education, net total expenditure, capital liabilities and industry [43]. Neighborhood social disparity was coded as 1 when participants lived in a deprived environment with a low FCC score relative to their own SES. Disparity was coded as 0 when participants lived in an environment matching with their individual SES (i.e. the neighborhood had a high FCC score and individual had high SES or reversed, low FCC and low SES).

### Genotyping and Genetic Risk Scores

Blood samples were taken when adolescents were 16 years old. The DNA extractions, sample quality controls, biobank up-keeping and aliquotting were performed in the National Public Health Institute, Biomedicum Helsinki, Finland. The rs900400 and rs9883204 single nucleotide polymorphisms (SNPs) near *LEKR* and *CCNL1* and in the *ADCY5* were genotyped (n = 5369) by Taqman allelic discrimination. No deviation (p≥0.05) from Hardy-Weinberg equilibrium was observed [12]. Success rate in genotyping was 0.96 for both SNPs. For the analyses, we categorized the genetic variants into two classes: 0 and at least 1 risk allele.

### Outcome Measures

Data on infant birth size, i.e. birthweight (in kg), head circumference (in cm) and birth length (in cm), were collected at birth by trained medical staff according to standardized procedure, entered into the medical records, and transferred onto the study forms. Ponderal index was calculated using the standard formula [birth weight (kg)/birth length (m^3^)].

### Covariates

Gestational age was calculated from the date of the last menstrual period (in 16%) or ultrasound examination (in 84% of the pregnant women). Maternal pre-pregnancy BMI was calculated using standard formula [kg/m^2^]. Information on smoking (nonsmoker = 0; smoker = 1), alcohol consumption (no alcohol consumed = 0; alcohol consumed = 1) and parity were taken from the self-report questionnaires during pregnancy. Blood pressure (BP) during pregnancy was classified as gestational hypertension (BP≥140/90 in the absence of proteinurea after the 20^th^ gestational week), pre-eclampsia (BP≥140/90 with proteinurea after the 20^th^ gestational week), chronic hypertension (on anti-hypertensive medication due to pre-existing hypertensive disorder or blood pressure of ≥140/90 before the 20^th^ week of gestation), superimposed pre-eclampsia (chronic hypertension with proteinurea), proteinuria (BP≥140, diastolic <90 with proteinurea, or diastolic ≥90 and systolic<140 with proteinurea), and normotensive. Protein urea was tested using a urinary dip-stick test (≥0.3 g/L). Oral glucose tolerance test (OGTT) was used as a method of screening mothers for gestational diabetes mellitus according to Finnish national guidelines, between the 26 and 28 gestational weeks. Screening was indicated in the case of glucosuria, prior gestational diabetes mellitus, suspected fetal macrosomia, previous macrosomic infant (birthweight >4500 g), maternal pre-pregnancy body mass index greater than 25 kg/m^2^ and age greater than 40 years. OGTT was performed using oral glucose load of 75 g after overnight fasting; upper ranges were 5.5, 11.0, and 8.0 mmol/L at fasting, 1 hour and 2 hours post glucose load. A single abnormal value in the OGTT was considered pathological and diagnoses of gestational diabetes mellitus made [44].

### Statistical Analysis

We calculated frequencies and percentages for descriptive analysis of the maternal and infant demographic and anthropometric measures. Using graphical tools we examined the distributions for normality and linearity, and used Pearson and Spearman correlations for continuous and categorical data, respectively, to examine multicollinearity. We used Chi square test statistics to test for unadjusted associations between social stress and categorized birth outcomes and maternal factors (as shown in Table S1).

To determine the adjusted association between social stress and birth size we conducted multiple linear regression analysis and adjusted the model for several *a priori* selected well known predictors of birth size and exposures that may confound the associations i.e. gestational age, maternal pre-pregnancy body mass index (BMI), smoking, alcohol consumption, parity, gestational diabetes and hypertensive disorders during pregnancy. The analyses were performed for males and females together (adjusted for sex) and separately by sex. We examined the additive effect of social stress and carrying at least one risk allele by comparing the reference group who had neither social stress or risk allele (coded as 0) with the following groups who either: 1) carried at least one risk allele only, 2) experienced social stress only, and 3) had both social stress and at least one risk allele. The P-value for trend across the exposure categories was calculated.

Tests were two-tailed and the level of significance set at 0.05. We did not use correction for multiple testing due to *a priori* set hypotheses, and analytical strategies. We used the version 9.1 of the SAS system for windows (SAS Institute Inc, Cary, NC) for statistical analyses.

## Results

### Cohort Characteristics

In the NFBC 1986 cohort 24.5% of mothers experienced at least one type of adversity during pregnancy, 3.6% two and 0.3% three adversities. Of the women experiencing at least one adversity, 36% had low education (<11years), 5% were single parents, and 4% were young (<20 years) at the time of delivery. Table S1 shows the association analyses of social stress (composite variables) with birth outcomes and maternal characteristics. Mothers experiencing any social adversity were more likely to deliver before the 37^th^ gestational week (*P*<0.0001), to be smokers (*P*<0.0001), and had higher maternal pre-pregnancy BMI (*P*<0.0001). Mothers from lower SES were also more likely to be smokers (*P*<0.0001) to be multipara (*P*<0.0001), have a high pre-pregnancy BMI (*P*<0.0001) and report at least one adversity (*P*<0.0001) [data not shown]. Single-parenthood was the component of the social adversity composite that showed the strongest association with birth size in comparison to infants from two-parent families in a mutually adjusted analysis (data not shown). This association between single parenthood and birth size was significant in males but not in females (males: birthweight −114 g (95%CI = −178.08, −50.13), birth length −0.40 cm (95%CI = −0.67, −0.14), head circumference −0.34 cm (95%CI = −0.53, −0.15); females: birthweight −16.87 g (95%CI = −4.15, 76.89) birth length 0.10 cm (95%CI = −0.16, 0.35 ), head circumference 0.03 cm (95%CI = −0.15, 0.21). Table S2 provides the distribution of the birth size outcomes according to genotype; with a frequency of ca 1582 (29.9%) for at least one risk allele in rs900400, and 4668 (96.6%) in rs9883204.

### Multiple Regression Analyses

Tables 1, 2, 3 and 4 present the results of the multiple regression analysis examining the association between social stress and birth size as well as the additive effects of the birthweight-lowering alleles.

**Table 1:** Multiple Linear Regression Analysis (GLM) of the association between social adversity and birth size [birth weight (g), length (cm), head circumference (cm) and ponderal index] in the whole NFBC86 Cohort (*n* = 9135) and stratified by sex.

	mean difference (95%CI) P-value											
	Birthweight (g)			Birth length(cm)			Head circumference(cm)			Ponderal index(kg/m^3^)		
Exposure:	*n* *(Yes/No)	Unadjusted	Adjusted**	*n* *(Yes/No)	Unadjusted	Adjusted**	*n* *(Yes/No)	Unadjusted	Adjusted**	*n* *(Yes/No)	Unadjusted	Adjusted**
ALL:^ r^												
Social Adversity^ r^ (yes, no[ref])	2278/5857	−76.3	−40.4	2265/5812	−0.32	−0.14	2238/5743	−0.17	−0.09	2265/5812	−0.01	−0.009
		(−100.0, −52.6)	(−61.4, −19.5)		(−0.42, −0.22)	(−0.23, −0.05)		(−0.24, −0.10)	(−0.15, −0.02)		(−0.02, −0.001)	(−0.02, −0.002)
		*<0.0001*	*0.0002*		*<0.0001*	*0.002*		*<0.0001*	*0.007*		*0.03*	0.10
MALES:												
Social Adversity (yes, no[ref])	1164/2990	−72.2	−39.3	1158/2970	−0.27	−0.12	1148/2941	−0.19	−0.09	1158/2970	−0.01	−0.01
		(−105.2, −39.2)	(−68.8, −9.9)		(−0.41, −0.14)	(−0.24, 0.0009)		(−0.29, −0.10)	(−0.19, −0.007)		(−0.03, 0.002)	(−0.02, 0.005)
		*<0.0001*	*0.009*		*<0.0001*	0.051		*<0.0001*	*0.04*		*0.01*	0.20
FEMALES:												
Social Adversity (yes, no[ref])	1114/2867	−79.5	−41.8	1107/2842	−0.36	−0.16	1090/2802	−0.14	−0.09	1107/2842	−0.01	−0.009
		(−112.9, −46.0)	(−71.6, −12.0)		(−0.49, −0.22)	(−0.29, −0.04)		(−0.23, −0.05)	(−0.18, −0.006)		(−0.03, 0.004)	(−0.03, 0.008)
		*<0.0001*	*0.006*		*<0.0001*	*0.01*		*0.003*	0.07		*0.15*	0.30

* *n* in the adjusted model.

** Adjusting for gestational age, maternal smoking, maternal alcohol consumption, parity, maternal pre-pregnancy BMI, gestational diabetes and hypertension during pregnancy. **^r^** Additionally adjusting for sex.

**Table 2:** Mean differences (95% confidence intervals, CI) in birth size as predicted by the additive effects of social Adversity and at least one risk allele (*CCNL1/LEKR1*- rs900400).

	Mean difference (95% CI) P value											
	Birth weight (g)			Birth Length(cm)			Head circumference (cm)			Ponderal index (kg/m^3^)		
Exposure:	*n* *	Unadjusted	Adjusted**	*n* *	Unadjusted	Adjusted**	*n* *	Unadjusted	Adjusted**	*n* *	Unadjusted	Adjusted**
Neither adversity nor riskallele [ref]	1546			1540			1517			1540		
At least one risk allele only	1873	−55.6	−76.6	1856	−0.03	−0.13	1836	−0.10	−0.16	1856	−0.35	−0.37
		(−88.6, −22.6)	(−105.0, −48.3)		(−0.17, 0.11)	(−0.25, −0.02)		(−0.19, −0.01)	(−0.25, −0.07)		(−0.50, −0.20)	(−0.52, −0.22)
		*0.001*	*<0.0001*		0.68	*0.03*		*0.03*	*0.0003*		*<0.0001*	*<0.0001*
Social adversity only	535	−106.4	−74.0	530	−0.34	−0.20	526	−0.25	−0.18	530	−0.29	−0.27
		(−153.8, −59.1)	(−115.8, −32.1)		(−0.54, −0.14)	(−0.38, −0.02)		(−0.39, −0.12)	(−0.31, −0.05)		(−0.50, −0.08)	(−0.50, −0.05)
		*<0.0001*	*0.0005*		*0.0008*	*0.03*		*0.0002*	*0.005*		*0.008*	*0.02*
Both adversity and at leastone risk allele	666	−106.8	−118.4	665	−0.29	−0.30	655	−0.19	−0.23	665	−0.41	−0.47
		(−151.1, −62.6)	(−156.9, −79.9)		(−0.48, −0.11)	(−0.46, −0.14)		(−0.32, −0.06)	(−0.35, −0.11)		(−0.61, −0.21)	(−0.67, −0.26)
		*<0.0001*	*<0.0001*		*0.002*	*0.0002*		*0.003*	*0.0001*		*<0.0001*	*<0.0001*
*P value for trend*			*<0.0001*			*0.0001*			*<0.0001*			*<0.0001*

* *n* in the adjusted model,

** controlling for gestational age, maternal smoking, maternal alcohol consumption, parity, maternal pre-pregnancy BMI, sex, gestational diabetes and hypertension during pregnancy.

**Table 3:** Multiple Linear Regression Analysis (GLM) of the association between neighborhood social disparity and birth size [birth weight (g), length (cm), head circumference (cm) and ponderal index] in the whole NFBC86 Cohort (*n* = 9135) and stratified by sex.

	mean difference (95%CI) P-value											
	Birthweight (g)			Birth length(cm)			Head circumference(cm)			Ponderal index(kg/m^3^)		
Exposure:	*n* * (Yes/No)	Unadjusted	Adjusted**	*n* * (Yes/No)	Unadjusted	Adjusted**	*n* * (Yes/No)	Unadjusted	Adjusted**	*n* * (Yes/No)	Unadjusted	Adjusted**
ALL:^ r^												
Neighborhood social disparity vs none [ref]	4404/3515	−5.7	−28.8	4370/3492	−0.06	−0.12	4327/3443	0.02	−0.02	4370/3492	0.003	−0.004
		(−27.8, 16.4)	(−47.7, −10.0)		(−0.15, 0.03)	(−0.20, −0.05)		(−0.04, 0.08)	(−0.08, 0.04)		(−0.007, 0.01)	(−0.02, 0.006)
		0.61	*0.003*		0.19	*0.003*		0.51	0.39		0.51	0.39
MALES:												
Neighborhood social disparity vs none [ref]	2261/1783	−5.3	−33.9	2246/1773	−0.12	−0.18	2230/1751	−0.0009	−0.07	2246/1773	0.01	0.002
		(−36.0, 25.5)	(−60.7, −7.0)		(−0.24, 0.01)	(−0.30, −0.08)		(−0.09, 0.09)	(−0.15, 0.01)		(−0.003, 0.03)	(−0.01, 0.02)
		0.74	*0.01*		0.07	*0.0009*		0.98	0.10		0.11	0.79
FEMALES:												
Neighborhood social disparity vs none [ref]	2143/1732	−6.5	−24.3	2124/1719	−0.004	−0.05	2097/1692	0.04	0.02	2124/1719	−0.005	−0.01
		(−37.8, 24.9)	(−50.9, 2.2)		(−0.13, 0.12)	(−0.16, 0.06)		(−0.04, 0.13)	(−0.06, 0.10)		(−0.02, 0.01)	(−0.03, 0.004)
		0.69	0.07		0.95	0.36		0.34	0.66		0.53	0.14

* *n* in the adjusted model.

** Adjusting for gestational age, maternal smoking, maternal alcohol consumption, parity, maternal pre-pregnancy BMI, gestational diabetes and hypertension during pregnancy. **^r^** dditionally adjusting for sex. Neighborhood social disparity =  living in an environment different to individual SES.

**Table 4:** Mean differences (95% confidence intervals, CI) in birth size as predicted by the additive effects of neighborhood social disparity and at least one risk allele (*CCNL1/LEKR1*- rs900400).

	Mean difference (95% CI) P value											
	Birth weight (g)			Birth Length(cm)			Head circumference (cm)			Ponderal index (kg/m^3^)		
Exposure:	*n* *	Unadjusted	Adjusted**	*n* *	Unadjusted	Adjusted**	*n* *	Unadjusted	Adjusted**	*n* *	Unadjusted	Adjusted**
Neither disparity nor riskallele [ref]	875			871			858			871		
At least one risk allele only	1044	−39.7	−63.5	1038	−0.006	−0.10	1024	−0.11	−0.17	1038	−0.34	−0.35
		(−84.1, 4.6)	(−101.4, −25.6)		(−0.19, 0.18)	(−0.26, 0.05)		(−0.23, 0.02)	(−0.30, −0.06)		(−0.54, −0.14)	(−0.56, −0.15)
		0.07	*0.001*		0.95	0.20		0.10	*0.003*		*0.0008*	*0.0007*
Social disparity only	1158	−11.8	−35.5	1151	−0.12	−0.16	1137	−0.06	−0.12	1151	0.02	−0.05
		(−55.2, 31.5)	(−72.4, 2.0)		(−0.30, 0.06)	(−0.32, 0.007)		(−0.19, 0.06)	(−0.24,−0.01)		(−0.17, 0.22)	(−0.25, 0.15)
		0.59	0.06		0.18	*0.04*		0.30	*0.03*		0.83	0.65
Both disparity and at leastone risk allele	1427	−54.7	−108.5	1415	−0.14	−0.31	1403	−0.12	−0.25	1415	−0.24	−0.37
		(−96.3, −13.0)	(−144.1, −72.9)		(−0.31, 0.04)	(−0.46, −0.16)		(−0.24, −0.006)	(−0.35, −0.14)		(−0.43, −0.05)	(−0.56, −0.17)
		*0.01*	*<0.0001*		0.12	*<0.0001*		*0.04*	*<0.0001*		0.01	*0.0002*
*P value for trend*			*<0.0001*			*<0.0001*			*<0.0001*			*0.008*

* *n* in the adjusted model,

** controlling for gestational age, maternal smoking, maternal alcohol consumption, parity, maternal pre-pregnancy BMI, sex, gestational diabetes and hypertension during pregnancy.

Table 1 shows the association between social adversity and birth size after adjusting for gestational age, maternal smoking, alcohol consumption, pre-pregnancy BMI, parity, gestational diabetes and hypertensive disorders during pregnancy. After adjustment infants of mothers who experienced at least one social adversity had smaller birth sizes as compared to infants of mothers with no adversity. Once stratified by sex, this difference remained statistically significant in males and females for birthweight, but only in females for birth length (−0.16 cm, 95%CI −0.29, −0.04) and in males for head circumference, (−0.09 cm, 95%CI −0.19, −0.007) respectively.

When adding genetic variance into the analysis (Table 2); compared to the reference category zero, i.e. no social adversity nor birthweight-lowering risk allele at rs900400, belonging to categories one, two or three (as described above) was associated with smaller birth size. The association with birth size was magnified in category three containing both social adversity and risk allele at rs900400 compared to the results for social adversity only and carrying one risk allele only; with reduction in birthweight by a total of −118 g (95%CI = −156.9, −79.9), birth length of −0.30 cm (95%CI =  −0.46, −0.14), head circumference by −0.23 cm (95%CI =  −0.35, −0.11), and ponderal index of −0.47 kg/m^3^(95%CI =  −0.67, −0.26). The P-value for trend for effect sizes by exposures categories was significant for all birth size outcomes. When stratified by sex (Table S3), this difference in birth size though not always reaching statistical significance due to reduced sample size, was more prominent in females. The results for rs9883204 (Table S4) showed no association across the categories, which may be attributed to insufficient sample size in the fully adjusted analyses.

Neighborhood social disparity was associated with smaller birth size with a difference in birthweight of −28.8 g (95% CI −47.7, −10.0) and birth length of −0.12 cm (95% CI −0.20, −0.05) in the adjusted model (Table 3). In the stratified model, the association of neighborhood social disparity with birth size was statistically significant in males only. When examining the association of neighborhood social disparity and genetic vulnerability (rs900400), compared to the reference group i.e. category zero, belonging to categories one, two or three was associated with smaller birth size (Table 4). However, the results did not reach statistical significance in all categories, though the P-values for trend for effect sizes by exposures categories were significant for all birth size outcomes. Here again, carrying at least one rs900400 birth weight lowering risk allele magnified the association with smaller birth size and neighborhood disparity. The association analyses for rs9883204 were non-significant.

## Discussion

Many human diseases stem from complex interplay between environmental and individual susceptibility. In our study we examined how and whether specific genetic susceptibility modulates the association with adverse outcomes from environmental exposure such as social stress. Our results show that maternal social stress during pregnancy, both at the individual and neighborhood levels, was associated with smaller infant birth size and that carrying the birthweight-lowering rs900400 C allele located near *CCNL1/LEKR* magnified this association. These results provide support for the hypothesis that an individual with a genetic or other biological risk is more vulnerable to environmental adversity. Though the magnitude of the reduction in birth size attributable to variants in genotype is of significant proportion (44 g) we can only stipulate a trend from these results. Though both social stress and the birth weight lowering allele rs9883204 at *ADCY5* [*12*] (in larger samples) are associated with smaller birth size, we were not able to report any significant additive effects on birth size. This is most likely due to limitations due to reduced sample size in our cohort as we performed complete case analysis. Larger sample studies are needed in order to determine the underlying social and biological pathways for the additive effect, genetic liability and environmental adversity have on fetal development.

Though variants near *CCNL1/LEKR* are linked to lower birth weight, the biology behind this association is still unclear [12]. Insulin is one of the most important fetal growth hormone, and the fetal insulin hypothesis suggests that genetic variants in glucose and insulin metabolism may affect fetal growth [45], [46]. However the *CCNL1/LEKR* has not yet been linked to either with type 2 diabetes or adult glycemic traits. On the contrary, a recent study has shown an association between the C-allele of the rs900400 located near *CCNL1/LEKR1* and increased insulin response to oral glucose stimulation in non-diabetic individual [47].

The impact of maternal social stress had on infant birthweight is comparable to mothers smoking two cigarettes per day during pregnancy [48], [49]. This highlights the importance of maternal social stress, both at individual and neighborhood levels, as an indicator for increased risk for lower infant birth size and consequent development of disease later in life.

Small head circumference is linked with poor developmental and cognitive outcomes in the offspring [3], [50], 1 cm increase in head circumference represents11% brain volume at term [51]–[53]. In our study maternal social stress was associated with both smaller infant head circumference and birth length; however, the differences were small. Measurement error cannot be excluded, *e.g.* head circumference is a measure of occipital-frontal circumference and is subject to some degree of unreliability. However, measurement error is unlikely to be systematically associated with social stress, thus should be considered as random variance and evenly distributed by exposure status.

Previous studies have shown male fetuses are more vulnerable to intrauterine insults [54]. The sexual dimorphic association with birth size we report here indicates differential response to fetal environmental factors between the sexes. This may help explain some of the gender differences involved in the cascade of development of diseases as seen later in life.

We hypothesize that maternal perceived stress as one of the biological pathways through which social stress affects birth size. Perceived stress has been linked to changes in maternal stress hormone levels (i.e. cortisol) during pregnancy [55]. Social adversity as defined here is a measure of stress related to biological and individual adversity, rather than just as financial and economic adversity. Maternal stress during pregnancy is known to be associated with physiological and cognitive outcomes in offspring [56]–[58]. Animal studies have shown that increased levels of maternal glucocorticoids, which are a major component of the stress response, are associated with smaller birth size in offspring [59], [60], [60]–[65]. The fetus may react to stress in an analogous manner as adults, *i.e.* with increased levels of fetal cord cortisol during late pregnancy [61]–[63]. However, studies have yet to show an increase in fetal cortisol levels due to maternal stress [66]. Other biological pathways that may link maternal stress to birth size, reduced placental blood flow and maternal diet (over and under nutrition), have yet to be tested [67]–[69].

Our study also highlights the importance of using three indicators of social adversity in the clinical setting in identifying pregnant women at risk for poor birth outcomes. The factors used to define social adversity are indicative of potential economic hardship and biological risk [24]. We showed that women experiencing some social adversity were more likely to smoke and deliver prematurely, risk factors known to predict small birth size [48], [49]. Importantly, the presence of only a single index of adversity was enough to result in lower birthweight, with maternal single marital status having the strongest association. Social adversity is also important as it may not only affect fetal development, but also influence postnatal development in terms of poor maternal resources.

The association of neighborhood social disparity with smaller birth size is likely to be explained by stress related to access to amenities. In Finland, despite uniformly distributed tax-paid health care, many individuals in rural areas, may require long distance travel to access *e.g.* health care and other amenities. Interestingly, though these types of neighborhood deprivations are small as compared to other industrialized nations, they still accounted for clinically significant differences in birth size.

Strengths of this study include the prospective data collection with extensive maternal and infant demographic and medical information. The study population is known to be genetically homogenous consisting of white Caucasians, therefore, reducing bias introduced by ethnicity. Moreover, we were able to adjust for major confounders, which has not been possible at this scale in previous studies. The study had, however, limited statistical power to report the additive effects of genetic variants on birth size with higher precision, or to test for any interactions between the genetic variants and social stress. These aspects should ideally be addressed in larger meta-analyses combining several studies, but the availability of such data has become a key issue. Another limitation is that we hypothesized that the objective measures of social stress employed in this study correspond to the biological stress response *e.g.* in hypothalamic pituitary adrenal-axis functioning. In this regard, it would have been a strength to have maternal blood cortisol samples available. However, cortisol in relation to perceptions of stress is fraught with measurement difficulties [70]. Nonetheless, the indices that we used have been previously correlated with perceived stress [71]–[74].

This study shows that genetic susceptibility magnified the association between social stress, (both at the individual and neighborhood levels) and birth size. The fact that social stress was associated with smaller birth size even in a society where there is relatively little social inequality as compared with other high-income countries and where tax–paid health care is universally available, is alarming and highlights the strength of the association. Moreover, social stress was a stronger predictor of birth size than having birthweight-lowering alleles, which emphasizes the use of indicators of social stress in clinical settings. It is promising that the addition of the genetic variants made a significant additional contribution which calls for further work in identifying groups of genetic variants and their interaction with environment.

## Supporting Information

Table S1 Chi-square tests of associations between maternal characteristics, birth outcomes and social stress in the whole NFBC86 Cohort.
(DOCX)

Table S2 Genotype frequency by birth size outcomes including means (**±**SD).
(DOCX)

Table S3 Mean differences (95% confidence intervals, CI) in birth size as predicted by the additive effects of social adversity and at least one risk allele (*CCNL1/LEKR1*- rs900400) stratified by sex.
(DOCX)

Table S4 Mean difference (95% confidence intervals, CI) in birth size as predicted by the additive affects of maternal social adversity and at least one risk allele (*ADCY5*-rs9883204).
(DOCX)
