# Supplementary material for: The Interplay of Variants Near LEKR and CCNL1 and Social Stress in Relation to Birth Size
Source: PLoS One. 2012 Jun 7;7(6):e38216. doi: 10.1371/journal.pone.0038216 (PMC3369922; doi:10.1371/journal.pone.0038216)
Supplement: Table S1 — Chi-square tests of associations between maternal characteristics, birth outcomes and social stress in the whole NFBC86 Cohort. (DOCX) [file pone.0038216.s001.docx]

**Table S1** Chi-square tests of associations between maternal characteristics, birth outcomes and social stress in the whole NFBC86 Cohort

|  |  | **Social Stress** | |  | |
| --- | --- | --- | --- | --- | --- |
|  |  | **Social adversity n(%)*** | | **Neighborhood social disparity n(%)** | |
| **Maternal Characteristics:** | | **Yes** | **No** | **Yes** | **No** |
| Smoking | Non-smoker | 1650(64.1) | 5422(83.0) | 1351(68.2) | 5715(80) |
|  | Smoker | 913(35.5) | 1080(16.5) | 576(29.1) | 1418(19.8) |
|  | *Missing* | *11(0.4)* | *30(0.5)* | *54(2.7)* | *15(0.2)* |
|  | *p value* | *<0.0001* |  | *<0.0001* |  |
|  |  |  |  |  |  |
| Alcohol consumption | Non-drinker | 2149(83.5) | 5473(83.8) | 1532(77.3) | 6084(85.1) |
|  | Drinker | 337(13.1) | 732(11.2) | 272(13.7) | 797(11.2) |
|  | *Missing* | *88(3.4)* | *327(5.0)* | *177(8.9)* | *267(3.7)* |
|  | *p value* | *0.02* |  | *<0.0001* |  |
|  |  |  |  |  |  |
| Parity | Nulliparaous | 932(36.2) | 2154(33.0) | 882(44.5) | 2204(30.8) |
|  | Multiparous | 1637(63.6) | 4369(66.9) | 1066(53.8) | 4935(69.0) |
|  | *missing* | *5(0.2)* | *9(0.1)* | *33(1.7)* | *9(0.1)* |
|  | *p value* | *0.003* |  | *<0.0001* |  |
|  |  |  |  |  |  |
| BMI | Low (≤18.5) | 285(11.1) | 590(9.0) | 277(14) | 625(8.7) |
|  | Normal(>18.5, ≤ 25) | 1780(69.2) | 4938(75.6) | 1412(71.3) | 5304(74.2) |
|  | Overweight (>25, ≤30) | 382(14.8) | 791(12.1) | 139(7.02) | 647(9.1) |
|  | Obese(>30) | 127(4.9) | 213(3.3) | 41(2.1) | 206(2.9) |
|  | *Missing* | *0* | *0* | *112(5.7)* | *366(5.1)* |
|  | *p value* | *<0.0001* |  | *<0.0001* |  |
|  |  |  |  |  |  |
|  |  |  |  |  |  |
|  |  |  |  |  |  |
|  |  |  |  |  |  |
|  |  |  |  |  |  |
| Hypertensive disorder | Gestational hypertension | 89(3.5) | 183(2.8) | 116(3.0) | 146(3.0) |
|  | Pre-eclampsia | 61(2.6) | 121(1.9) | 85(2.2) | 91(1.9) |
|  | Chronic hypertension | 68(2.6) | 143(2.2) | 66(1.7) | 127(2.7) |
|  | Superimposed pre-eclampsia | 33(1.3) | 55(0.8) | 29(0.8) | 48(1.0) |
|  | Proteinurea | 327(12.7) | 753(11.5) | 456(11.8) | 559(11.7) |
|  | Normotensive | 1932(75.1) | 5179(79.3) | 3050(79.0)) | 3692(77.6) |
|  | *Missing* | *64(2.5)* | *98(1.5)* | *59(1.5)* | *97(2.0)* |
|  | *p value* | *0.005* |  | *0.04* |  |
|  |  |  |  |  |  |
| Gestational Diabetes | Pre-pregnancy diabetes mellitus | 5(0.2) | 19(0.3) | 9(0.2) | 14(0.3) |
|  | Abnormal OGTT | 49(1.9) | 88(1.4) | 62(1.6) | 70(1.5) |
|  | OGTT not performed despite indicated | 589(22.9) | 1316(20.2) | 697(18.1) | 1147(24.1) |
|  | No OGTT neither indications | 1418(55.1) | 3942(60.4) | 2417(62.6) | 2815(59.1) |
|  | OGTT normal | 307(11.9) | 773(11.8) | 526(13.6) | 524(11.0) |
|  | *Missing* | *206(8.0)* | *394(6.0)* | *150(3.9)* | *190(4.0)* |
|  | *p value* | *0.0005* |  | *<0.0001* |  |
|  |  |  |  |  |  |
| **Birth Outcomes:** |  |  |  |  |  |
| Gestational age (gw) | <34 | 24(0.9) | 53(0.8) | 21(1.1) | 56(0.8) |
|  | 34-36 | 104(4.0) | 165(2.5) | 69(3.5) | 203(2.8) |
|  | 37-39 | 1110(43.1) | 2681(41.0) | 799(40.3) | 3001(42) |
|  | 40-42 | 1329(51.6) | 3624(55.5) | 1084(54.7) | 3875(54.2) |
|  | 43+ | 7(0.3) | 9(0.1) | 3(0.2) | 13(0.2) |
|  | *Missing* | *29(0.3)* | *5(0.1)* | *5* | *0* |
|  | *p value* | *0.0001* |  | *0.32* |  |
|  |  |  |  |  |  |
| Birthweight (g) | <1500 | 5(0.2) | 14(0.2) | 5(0.3) | 14(0.2) |
|  | 1500-2499 | 66(2.6) | 141(2.6) | 55(2.8) | 156(2.2) |
|  | 2500-2999 | 283(11.0) | 480(7.4) | 196(9.9) | 571(8) |
|  | 3000-3999 | 1750(68.0) | 4506(69) | 1359(68.6) | 4910(68.7) |
|  | ≥4000 | 470(18.3) | 1391(21.3) | 366(18.5) | 1497(20.9) |
|  | *Missing* | *0* | *0* | *0* | *0* |
|  | *p value* | *<0.0001* |  | *0.008* |  |
|  |  |  |  |  |  |
| Head circumference (cm) | ≤33 | 104(4.0) | 164(2.5) | 83(4.2) | 189(2.6) |
|  | 33-33.9 | 253(9.8) | 493(7.6) | 214(10.8) | 533(7.5) |
|  | 34-34.9 | 506(19.7) | 1200(18.4) | 411(20.8) | 1299(18.2) |
|  | 35-35.9 | 668(26.0) | 1803(27.6) | 523(26.4) | 1951(27.3) |
|  | 36-36.9 | 603(23.4) | 1608(24.6) | 412(20.8) | 1803(25.22) |
|  | 37-37.9 | 290(11.3) | 871(13.3) | 229(11.6) | 938(13.1) |
|  | ≥38 | 104(4.04) | 260(3.9) | 67(3.4) | 296(4.4) |
|  | *Missing* | *46(1.8)* | *133(2.0)* | *42(2.1)* | *139(1.9)* |
|  | *p value* | *<0.0001* |  | *0.0005* |  |
|  |  |  |  |  |  |
| Birth length(cm) | <47 | 109(4.2) | 208(3.2) | 93(4.7) | 229(3.2) |
|  | 47-48.9 | 336(13.1) | 631(9.7) | 243(12.3) | 726(10.2) |
|  | 49-52.9 | 1744(67.8) | 4513(69.1) | 1333(67.3) | 4936 (69.1) |
|  | 53-55.9 | 352(13.7) | 1085(16.6) | 287(14.5) | 1154(16.1) |
|  | ≥56 | 15(0.6) | 42(0.6) | 10(0.5) | 46(0.6) |
|  | *Missing* | *18(0.7)* | *53(0.8)* | *15(0.8)* | *57(0.8)* |
|  | *p value* | *<0.0001* |  | *0.0005* |  |

*Social adversity was defined as the sum of the following characteristics: young maternal age (1=< 20 years, 0=>20 years, low maternal education (1=≤11years, 0=>11years), and single marital status (1==unmarried, divorced or widowed, 0= married or cohabiting with the expectant father), OGTT=oral glucose tolerance test
