# Supplementary material for: The Interplay of Variants Near LEKR and CCNL1 and Social Stress in Relation to Birth Size
Source: PLoS One. 2012 Jun 7;7(6):e38216. doi: 10.1371/journal.pone.0038216 (PMC3369922; doi:10.1371/journal.pone.0038216)
Supplement: Table S2 — Genotype frequency by birth size outcomes including means (±SD). (DOCX) [file pone.0038216.s002.docx]

**Table S2** Genotype frequency by birth size outcomes including means (**±**SD)

|  | **Birth weight (g)** | |  | **Birth length(cm)** | |  | **Head circumference (cm)** | |  | **Ponderal index (kg/m^3^)** | |
| --- | --- | --- | --- | --- | --- | --- | --- | --- | --- | --- | --- |
|  | ***n*(%)** | **Mean (±SD)** |  | ***n* (%)** | **Means (±SD)** |  | ***n* (%)** | **Means (±SD)** |  | ***n* (%)** | **Means (±SD)** |
| *LEKR1/CCNL1* rs900400 |  |  |  |  |  |  |  |  |  |  |  |
| CC | 176 | 3657.05 |  | 174 | 51.03 |  | 172 | 35.31 |  | 174 | 27.54 |
|  | (3.33) | (477.45) |  | (3.32) | (2.00) |  | (3.32) | (1.37) |  | (3.32) | (2.20) |
|  |  |  |  |  |  |  |  |  |  |  |  |
| CT | 1406 | 3599.78 |  | 1394 | 50.63 |  | 1369 | 35.20 |  | 1394 | 27.64 |
|  | (26.60) | (547.04) |  | (26.58) | (2.29) |  | (26.44) | (1.52) |  | (26.58) | (2.37) |
|  |  |  |  |  |  |  |  |  |  |  |  |
| TT | 3703 | 3580.48 |  | 3677 | 50.54 |  | 3637 | 35.18 |  | 3677 | 27.65 |
|  | (70.07) | (513.01) |  | (70.11) | (2.13) |  | (70.24) | (1.44) |  | (70.11) | (2.33) |
| *ADCY5*  rs9883204 |  |  |  |  |  |  |  |  |  |  |  |
| CC | 3379 | 3580 |  | 3357 | 50.55 |  | 3321 | 35.17 |  | 3357 | 27.64 |
|  | (69.92) | (511.2) |  | (69.89) | (2.12) |  | (70.03) | (1.44) |  | (69.92) | (2.34) |
|  |  |  |  |  |  |  |  |  |  |  |  |
| CT | 1289 | 3599.2 |  | 1281 | 50.67 |  | 1260 | 35.22 |  | 1281 | 27.59 |
|  | (26.67) | (544.1) |  | (26.67) | (2.23) |  | (26.57) | (1.50) |  | (26.68) | (2.34) |
|  |  |  |  |  |  |  |  |  |  |  |  |
| TT | 165 | 3657.6 |  | 165 | 51.01 |  | 161 | 35.36 |  | 163 | 27.56 |
|  | (3.41) | (499.6) |  | (3.44) | (2.13) |  | (3.40) | (1.39) |  | (3.40) | (2.12) |
|  |  |  |  |  |  |  |  |  |  |  |  |

Risk allele = C
