# Supplementary material for: The Interplay of Variants Near LEKR and CCNL1 and Social Stress in Relation to Birth Size
Source: PLoS One. 2012 Jun 7;7(6):e38216. doi: 10.1371/journal.pone.0038216 (PMC3369922; doi:10.1371/journal.pone.0038216)
Supplement: Table S3 — Mean differences (95% confidence intervals, CI) in birth size as predicted by the additive effects of social adversity and at least one risk allele (CCNL1/LEKR1- rs900400) stratified by sex. (DOCX) [file pone.0038216.s003.docx]

**Table S3** Mean differences (95% confidence intervals, CI) in birth size as predicted by the additive effects of social adversity and at least one risk allele (*CCNL1/LEKR1*- rs900400) stratified by sex

|  | **Mean difference (95%CI) P-value** | | | | | | | | | | | |
| --- | --- | --- | --- | --- | --- | --- | --- | --- | --- | --- | --- | --- |
|  | **Birthweight (g)** | | | **Birth length(cm)** | | | **Head circumference(cm)** | | | **Ponderal index(kg/m^3^)** | | |
| **Exposure:** | ***n**** | **Unadjusted** | **Adjusted**** | ***n**** | **Unadjusted** | **Adjusted**** | ***n**** | **Unadjusted** | **Adjusted**** | ***n**** | **Unadjusted** | **Adjusted**** |
| MALES ONLY: |  |  |  |  |  |  |  |  |  |  |  |  |
| Neither adversity nor risk allele [ref] | 737 |  |  | 733 |  |  | 724 |  |  | 733 |  |  |
| At least one risk allele only | 942 | -44.06 | -66.8 | 935 | -0.005 | -0.08 | 923 | -0.06 | -0.14 | 935 | -0.32 | -0.39 |
|  |  | (-90.79, 2.67) | (-107.7, -25.8) |  | (-0.20, 0.19) | (-0.24, 0.09) |  | (-0.20, 0.07) | (-0.26, -0.01) |  | (-0.52, -0.11) | (-0.60, -0.17) |
|  |  | 0.07 | *0.001* |  | 0.96 | 0.39 |  | 0.35 | *0.03* |  | *0.003* | *0.0004* |
| Social adversity only | 246 | -100.6 | -97.13 | 244 | -0.38 | -0.33 | 243 | -0.22 | -0.18 | 244 | -0.21 | -0.25 |
|  |  | (-169.5, -31.8) | (-159.0, -35.3) |  | (-0.66, -0.09) | (-0.59, -0.08) |  | (-0.42, -0.03) | (-0.37, 0.005) |  | (-0.51, 0.10) | (-0.57, 0.07) |
|  |  | *0.004* | *0.002* |  | *0.009* | *0.01* |  | *0.03* | *0.06* |  | 0.18 | 0.13 |
| Both adversity and at least one risk allele | 333 | -71.3 | -85.9 | 332 | -0.09 | -0.11 | 327 | -0.11 | -0.13 | 332 | -0.41 | -0.50 |
|  |  | (-133.6, -9.0) | (-141.2, -30.6) |  | (-0.35, 0.16) | (-0.33, 0.12) |  | (-0.29, 0.07) | (-0.30, 0.04) |  | (-0.69, -0.14) | (-0.78, -0.21) |
|  |  | *0.03* | *0.002* |  | 0.47 | 0.37 |  | 0.23 | 0.14 |  | *0.003* | *0.0007* |
| *P value for trend* |  |  | *0.0002* |  |  | 0.16 |  |  | *0.02* |  |  | *0.0002* |
|  |  |  |  |  |  |  |  |  |  |  |  |  |
| FEMALES ONLY: |  |  |  |  |  |  |  |  |  |  |  |  |
| Neither adversity nor risk allele [ref] | 809 |  |  | 807 |  |  | 793 |  |  | 807 |  |  |
| At least one risk allele only | 931 | -73.57 | -86.9 | 921 | -0.10 | -0.19 | 913 | -0.17 | -0.18 | 921 | -0.35 | -0.36 |
|  |  | (-119.4, -27.7 ) | (-126.3, -47.4) |  | (-0.29, 0.09) | (-0.36, -0.02) |  | (-0.30, -0.05) | (-0.30, -0.06) |  | (-0.57, -0.14) | (-0.58, -0.14) |
|  |  | *0.002* | *<0.0001* |  | 0.31 | *0.03* |  | *0.007* | *0.003* |  | *0.001* | *0.001* |
| Social adversity only | 289 | -107.2 | -53.7 | 286 | -0.28 | -0.09 | 283 | -0.26 | -0.18 | 286 | -0.37 | -0.28 |
|  |  | (-171.4, -43.1) | (-110.5, 3.2) |  | (-0.55, -0.02) | (-0.33, 0.15) |  | (-0.44, -0.09) | (-0.35, -0.01) |  | (-0.67, -0.07) | (-0.60, 0.03) |
|  |  | *0.001* | 0.06 |  | *0.04* | 0.46 |  | *0.004* | *0.04* |  | *0.02* | 0.08 |
| Both adversity and at least one risk allele | 333 | -147.9 | -149.5 | 333 | -0.53 | -0.49 | 328 | -0.30 | -0.33 | 333 | -0.38 | -0.44 |
|  |  | (-209.6, -86.3) | (-203.4, -95.7) |  | (-0.79, -0.28) | (-0.72, -0.27) |  | (-0.46, -0.13) | (-0.49, -0.17) |  | (-0.67, -0.10) | (-0.74, -0.15) |
|  |  | *<0.0001* | *<0.0001* |  | *<0.0001* | *<0.0001* |  | *0.0006* | *<0.0001* |  | *0.009* | *0.004* |
| *P value for trend* |  |  | *<0.0001* |  |  | *0.0002* |  |  | *<0.0001* |  |  | *0.003* |

**n* in the adjusted model, **controlling for gestational age, maternal smoking, maternal alcohol consumption, parity, maternal pre-pregnancy BMI , sex, gestational diabetes and hypertension during pregnancy
