# Supplementary material for: The Interplay of Variants Near LEKR and CCNL1 and Social Stress in Relation to Birth Size
Source: PLoS One. 2012 Jun 7;7(6):e38216. doi: 10.1371/journal.pone.0038216 (PMC3369922; doi:10.1371/journal.pone.0038216)
Supplement: Table S4 — Mean difference (95% confidence intervals, CI) in birth size as predicted by the additive affects of maternal social adversity and at least one risk allele (ADCY5-rs9883204). (DOCX) [file pone.0038216.s004.docx]

**Table S4** Mean difference (95% confidence intervals, CI) in birth size as predicted by the additive affects of maternal social adversity and at least one risk allele (*ADCY5*-rs9883204)

|  | **Mean difference (95%CI) P-value** | | | | | | | | | | | |
| --- | --- | --- | --- | --- | --- | --- | --- | --- | --- | --- | --- | --- |
|  | **Birthweight (g)** | | | **Birth length(cm)** | | | **Head circumference(cm)** | | | **Ponderal index(kg/m^3^)** | | |
| **Exposure:** | ***n**** | **Unadjusted** | **Adjusted**** | ***n**** | **Unadjusted** | **Adjusted**** | ***n**** | **Unadjusted** | **Adjusted**** | ***n**** | **Unadjusted** | **Adjusted* *** |
|  |  |  |  |  |  |  |  |  |  |  |  |  |
| Neither adversity nor risk allele [ref] | 21 |  |  | 21 |  |  | 21 |  |  | 21 |  |  |
| At least one risk allele only | 624 | -48.2 | 39.8 | 614 | -0.33 | 0.08 | 609 | -0.06 | 0.25 | 614 | 0.09 | 0.26 |
|  |  | (-140.7, 44.3) | (-161.9, 241.5) |  | (-0.71, 0.06) | (-0.72, 0.89) |  | (-0.33, 0.20) | (-0.33, 0.83) |  | (-0.34, 0.51) | (-0.78, 1.31) |
|  |  | 0.31 | 0.70 |  | 0.09 | 0.84 |  | 0.63 | 0.40 |  | 0.69 | 0.62 |
|  |  |  |  |  |  |  |  |  |  |  |  |  |
| Social adversity only | 9 | 2.6 | 228.0 | 9 | -0.000 | 0.83 | 8 | 0.23 | 1.04 | 9 | -0.08 | 0.54 |
|  |  | (-183.7, 188.8) | (-133.7, 589.8) |  | (-0.77, 0.77) | (-0.61, 2.27) |  | (-0.30, 0.76) | (-0.04, 2.13) |  | (-0.93, 0.76) | (-1.34, 2.42) |
|  |  | 0.98 | 0.22 |  | 1.00 | 0.26 |  | 0.39 | 0.06 |  | 0.85 | 0.57 |
|  |  |  |  |  |  |  |  |  |  |  |  |  |
| Both adversity and at least one risk allele | 271 | -132.9 | -80.0 | 267 | -0.66 | -0.32 | 267 | -0.23 | 0.03 | 267 | -0.10 | -0.14 |
|  |  | (-228.2, -37.6) | (-286.3, 126.3) |  | (-1.05, -0.26) | (-1.14, 0.50) |  | (-0.50, 0.04) | (-0.56, 0.63) |  | (-0.54, 0.34) | (-1.21, 0.94) |
|  |  | *0.006* | 0.45 |  | *0.001* | 0.45 |  | 0.10 | 0.91 |  | 0.65 | 0.80 |
|  |  |  |  |  |  |  |  |  |  |  |  |  |
| *P value for trend* |  |  | *0.05* |  |  | 0.12 |  |  | 0.09 |  |  | 0.14 |

**n* in the adjusted model

**controlling for gestational age, maternal smoking, maternal alcohol consumption, parity, maternal pre-pregnancy BMI , sex, gestational diabetes and hypertension during pregnancy
